# Single cell analysis reveals a biophysical aspect of collective cell-state transition in embryonic stem cell differentiation

**DOI:** 10.1038/s41598-018-30461-2

**Published:** 2018-08-10

**Authors:** Kazuko Okamoto, Arno Germond, Hideaki Fujita, Chikara Furusawa, Yasushi Okada, Tomonobu M. Watanabe

**Affiliations:** 1 RIKEN Center for Biosystems Dynamics Research (BDR), 6-2-3 Furuedai, Suita, Osaka 565-0874 Japan; 2 WPI, Immunology Frontier Research Center, Osaka University, 3-1 Yamadaoka, Suita, Osaka 565-0871 Japan; 3 School of Science, the University of Tokyo, 7-3-1 Hongo, Bunkyo-ku, Tokyo 113-0033 Japan; 4 Graduate School of Frontier Bioscience, Osaka University, 1-3 Yamadaoka, Suita, Osaka 565-0871 Japan

## Abstract

In the stem cell research field, the molecular regulatory network used to define cellular states has been extensively studied, however, the general driving force guiding the collective state dynamics remains to be identified from biophysical aspects. Here we monitored the time-development of the cell-state transition at the single-cell and colony levels, simultaneously, during the early differentiation process in mouse embryonic stem cells. Our quantitative analyses revealed that cellular heterogeneity was a result of spontaneous fluctuation of cellular state and cell-cell cooperativity. We considered that the cell state is like a ball fluctuating on a potential landscape, and found that the cooperativity affects the fluctuation. Importantly, the cooperativity temporarily decreased and increased in the intermediate state of cell differentiation, leading to cell-state transition in unison. This process can be explained using the mathematical equation of flashing-ratchet behaviour, which suggests that a general mechanism is driving the collective decision-making of stem cells.

## Introduction

Mouse embryonic stem cells (mESCs), which can be isolated from the inner cell mass of an early-stage preimplantation blastocyst, are able to undergo self-renewal and to differentiate into any type of cell in the body^1‒3^. The gene regulatory network, built upon core transcription factors such as Nanog, Oct4 (also known as Pou5f1), and Sox2, maintains the pluripotency in mESCs and controls lineage specifications^4‒7^. Interestingly, mESC differentiation is orderly organized and/or synchronized in the inner cell mass^8^, as the ESCs collectively change their cellular states during the early developmental stage. The mechanisms by which the mESCs act in unison to fulfil their roles during development have been the topic of a long-running debate^9^.

Paracrine signalling networks of the cell layer have been potentially involved in the process of the collective differentiation. Leukaemia inhibitory factor (LIF), for example, is necessary for maintenance of pluripotency^10‒13^. The LIF signal enhances Nanog expression via the PI3K/AKT cascade and Oct4 expression via the JAK/STAT3 cascade^14,15^. The JAK/STAT3 cascade is also dependent on E-cadherin signalling, which is known to be generated from the contact between cells (i.e., cell-cell adhesion)^16,17^. Hence, the level of Nanog and/or Oct4 expression within a given cell is correlated with the E-cadherin expression levels of the neighbouring cells in the initial and intermediate stage of early differentiation. Another contributor to paracrine signalling is the fibroblast growth factor 4 (FGF4) /ERK pathway, which mediates a negative feedback loop^18‒20^. Along with these kinds of molecular mechanisms, the biophysical vantage point has also contributed to our comprehension of the big picture of collective behaviour.

The accumulation of experimental and theoretical evidence over the past 50 years has shown that the cell-state transition process during cell differentiation is guided by two major components: a deterministic component exerted by a complex regulatory network, and an intrinsic stochastic component^21^. The core transcription factors for the pluripotency maintenance mentioned above are a part of much more intricate networks involving protein–protein interactions^22,23^, microRNAs^24^, and epigenetic factors^25^. Moreover, the heterogeneity of gene expression, due to stochasticity at the transcription and translation levels, has been considered an intrinsically ‘noisy’ molecular process that plays a determining role in the stem cell fate^21^. In fact, the expression of core transcription factors of individual mESCs exhibits a characteristic bimodal distribution of high and low expression levels^26,27^. When each fraction of the bimodal population was isolated and used for further cultures, the parental bimodal distribution was reconstituted^28^. These experimental findings strongly suggest that cells fluctuate stochastically between two different states. Importantly, the bimodal distribution of gene expression of a transcription factor could be exhibited not only at cell level but also at colony level, suggesting the presence of two states – and possibility some collective response – at the colony level^29^.

Based on the above theoretical and experimental considerations, conceptual efforts have been made to find a general mechanism explaining how the deterministic and stochastic components combine and drive the cell-state transition during cell differentiation^9,21^. For an accurate and quantitative understanding of the regulation of stem cell fate, it would be invaluable to find such a general mechanism. A mathematical model considering the paracrine signalling networks via the FGF4/ERK pathway successfully reproduced the spatial heterogeneity observed in mESCs^29^. Along with FGF4 paracrine secretion, the LIF signal inhibits the self-activation of Nanog via the GRAB2/ERK signalling cascade, thereby enhancing Nanog heterogeneity^12,30^. Thus, the heterogeneity or bimodal distribution at the colony level is an important characteristic of stem cell differentiation. This phenotype results from the interaction of both stochastics and deterministic components, namely, intrinsic fluctuation and cell-cell cooperativity: the cells are intrinsically and spontaneously fluctuating their own states, and extrinsically regulating the neighbouring cells’ states within a colony. In spite of its crucial importance for the understanding of stem cell dynamics, there are still no experimental reports that quantitatively investigate the biophysical mechanism driving colonial heterogeneity.

We here demonstrate that the cell-state transition of mESCs occurs during the early differentiation stage by simultaneously monitoring the expression levels of Nanog and Oct4 at both the individual cell and colony levels. Quantitative analyses at single cell level unveiled how the heterogeneity of cells affects neighbouring cells in a colony and how it affects colonial phenotype. Specifically, our results revealed that the dynamic change of the cell-cell cooperativity causes the alteration of the attractor shape of the potential landscape on which a ball, describing the cell state, is rolling. The cell state was found to fluctuate based on cell-cell cooperativity, which can be translated into a mathematical equation that has been used to describe flashing ratchet-like behaviours. This flashing ratchet-like behaviour appears to be a likely general mechanism driving the collective behaviour of mESCs at multiple scales.

## Results

### Observation of the heterogeneity of mESCs at cell and colony levels

To monitor the cell-state transition during the differentiation process of mESC, we established a mESC line expressing two fluorescent proteins, Venus and mKate2, under the control of the Nanog and Oct4 promoters, respectively. The mESC clones expressed Venus and mKate2 at high levels in the presence of LIF, whereas the absence of LIF resulted in a decrease in the Venus and mKate2 fluorescence intensities, as previously reported^10,11^. The fluorescent intensity of the reporter system significantly replicated the bimodal distribution of high and low expression states in immunofluorescence (Fig. S3). We defined this decrease as a transition toward the differentiated state. To facilitate the monitoring of the timing of the cell-state transition, we purposely selected clones that exhibited a relatively slow differentiation process: approximately half of the selected cells showed a reduction in their fluorescence intensities after 7–8 days in the absence of LIF signalling (Supplementary Figs S1 and S2). Clusters of cells exhibiting high Venus fluorescence were obtained after a longer period of culture without passaging in the absence of LIF, indicating that this mESC line could do collective behaviour (Fig. S4).

**Figure 1:**
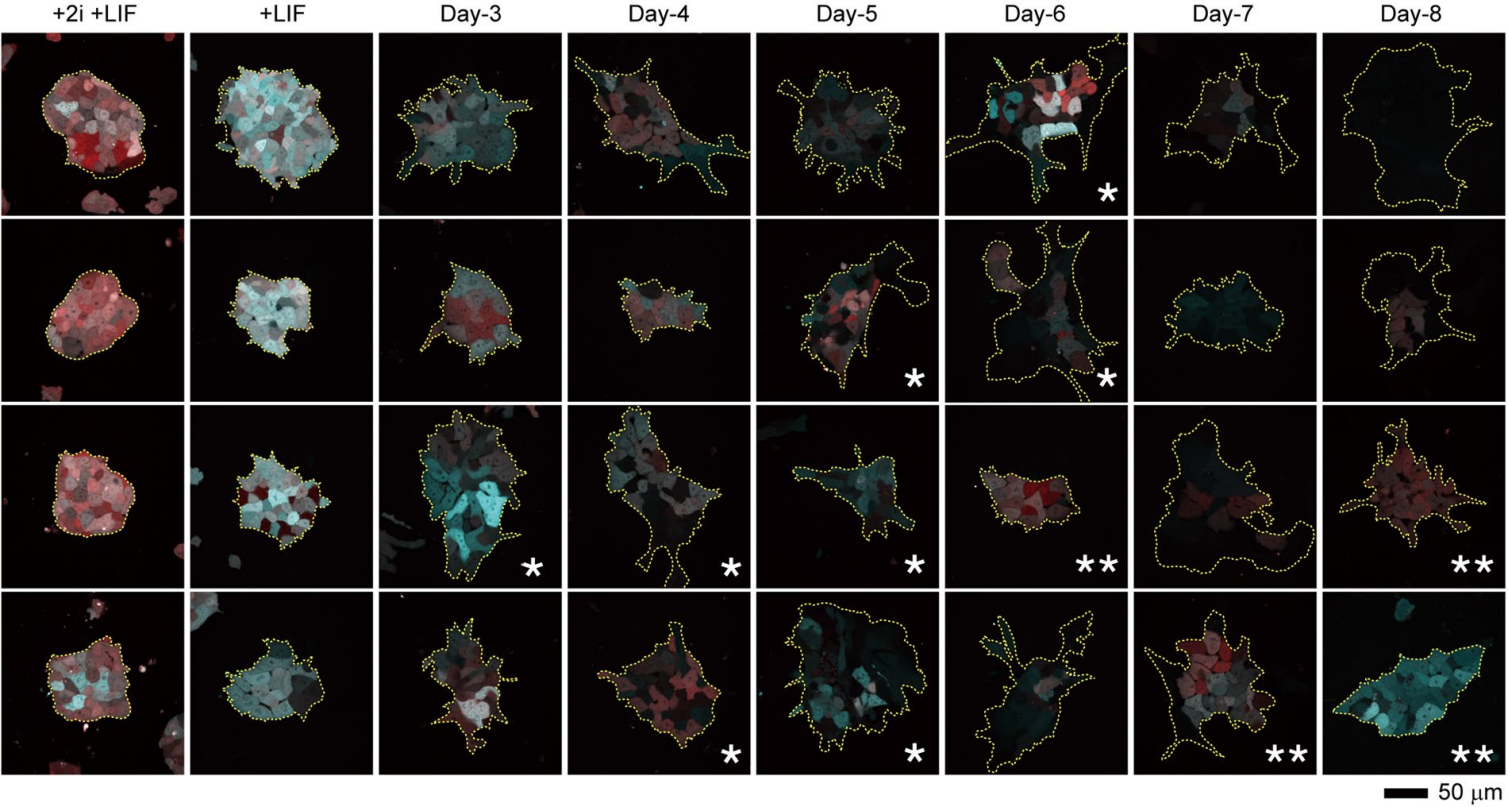
Observation of fluorescence heterogeneity at the single-cell and colony levels over the experimental period. Representative fluorescent images of Venus reporting Nanog expression (cyan) and mKate2 reporting Oct4 expression (red) of mESC colonies in the presence of LIF with 2i (+2i +LIF), in the presence of LIF only (+LIF), and in the absence of LIF from the 3^rd^ to 8^th^ days. Asterisks (*) indicate colonies exhibiting higher cellular heterogeneity. Double asterisks (**) indicate colonies maintaining their fluorescence at the later stage. Each image size is 211.76 × 211.76 μm^2^.

To monitor the development of the cell-state transition of mESCs over time, we acquired confocal fluorescence images of mESC colonies cultured in the presence of LIF (hereafter, termed +LIF) and during the early differentiation process of cells, from the 3^rd^ to the 8^th^ day in the absence of LIF (hereafter, termed Day-N, where N is the number of the days). In the +LIF condition, the cells and colonies were confirmed to show the highest fluorescence intensities, which decreased in the absence of LIF signalling. During the early stage of differentiation, microscopic observations revealed the stochastic nature of Nanog and Oct4 gene expression (Fig. 1). Strong cellular heterogeneity in expression levels was observed in some colonies between Day-3 and Day-6 (Fig. 1, asterisks). Some colonies maintained the relatively strong average fluorescence of the Nanog and Oct4 reporters between Day-6 and Day-8 (Fig. 1, double asterisks). These results confirmed the existence of heterogeneity not only at the single-cell level but also at the colony level in the present mESC line.

### Quantification of the heterogeneity at the single-cell and colony levels

**Figure 2:**
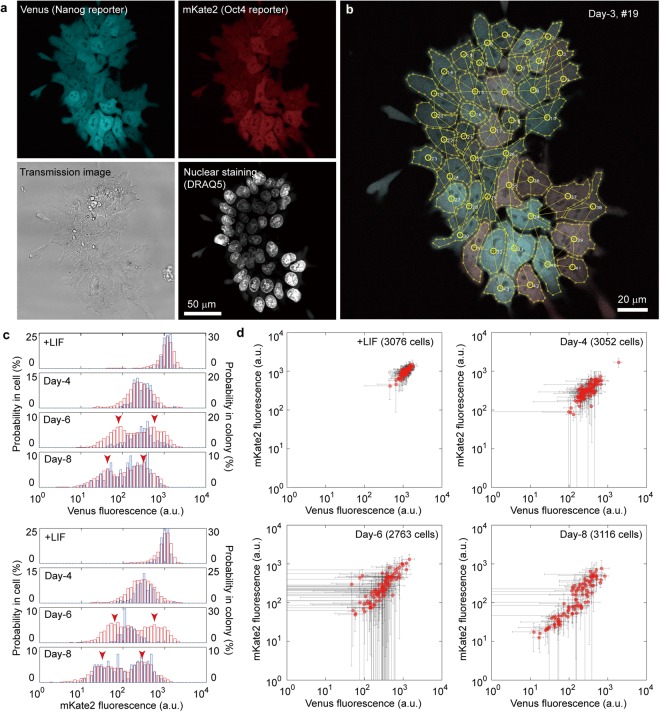
Quantitative analyses of the fluorescence heterogeneity at the single-cell and colony levels. (**a**) Fluorescence images of Venus (upper left), mKate2 (upper right), and nuclei stained with DRAQ5 (lower right), and a transmission light microscopy image of a colony (lower left). (**b**) Manual identification of single cells. Yellow broken lines indicate the cell peripheries, circles indicate the centroids of cells, and solid lines indicate the connections with adjacent cells. (**c**) Histograms of the average fluorescent intensity in each cell (red, left axis) and mean fluorescent intensity in each colony (blue, right axis). Arrowheads indicate the peaks of the bimodal distribution measured at the single-cell level. (**d**) Correlation plots of the mean intensity of Venus and mKate2 fluorescence in single colonies. Black lines indicate standard deviations in each colony.

Images of 100 colonies composed of approximately 30 cells per colony at 8 conditions were collected and analysed (total number of cells = 26,714, see Supplementary Table S1). Despite the availability of powerful software for automatic cell identification^31^, we had no choice but to identify the contour of individual cells by eye from the transmission microscope image using nuclear staining (Fig. 2a and b) owing to the large variation of cell morphologies and nuclear shapes at different stages of differentiation (Fig. 1). For each cell, fluorescence intensities were calculated from the average fluorescence of all pixels within the cell area. Histograms of the average fluorescence of Venus and mKate2 for individual cells exhibited a single population at Day-3, Day-4, and Day-5 (Figs 2c and S5), which shifted toward a bimodal distribution from Day-6 to Day-8 (Figs 2c and S5, arrowheads). There was no obvious difference between the results of Venus and mKate2.

For each colony, the mean and standard deviation of Venus and mKate2 fluorescence intensities were calculated from the average intensities of all single cells forming a given colony. Overall, the distribution pattern of the mean of fluorescence intensities in colonies was similar (Fig. 2c blue, and Fig. S6) to the aforementioned distribution of fluorescence intensities of individual cells (Fig. 2c red, and Fig. S5), with the exception that no bimodal distribution was observed at Day-6, but only from Day-8 (Fig. 2c, blue). Standard deviations, reflecting cellular heterogeneity in each colony, gradually increased from Day-3 to Day-6 and then decreased over the next two days (Figs 2d and S6, black lines). Attributing a different colour for each cell within a given colony enabled visualization of this temporal change in heterogeneity (Fig. S7). These results indicate a bimodal population representing two distinct cellular states whose pattern emerged around Day-6, and that this change in the distribution was delayed at the colony level, appearing at Day-8.

### Quantification of gene expression variation in colonies

**Figure 3:**
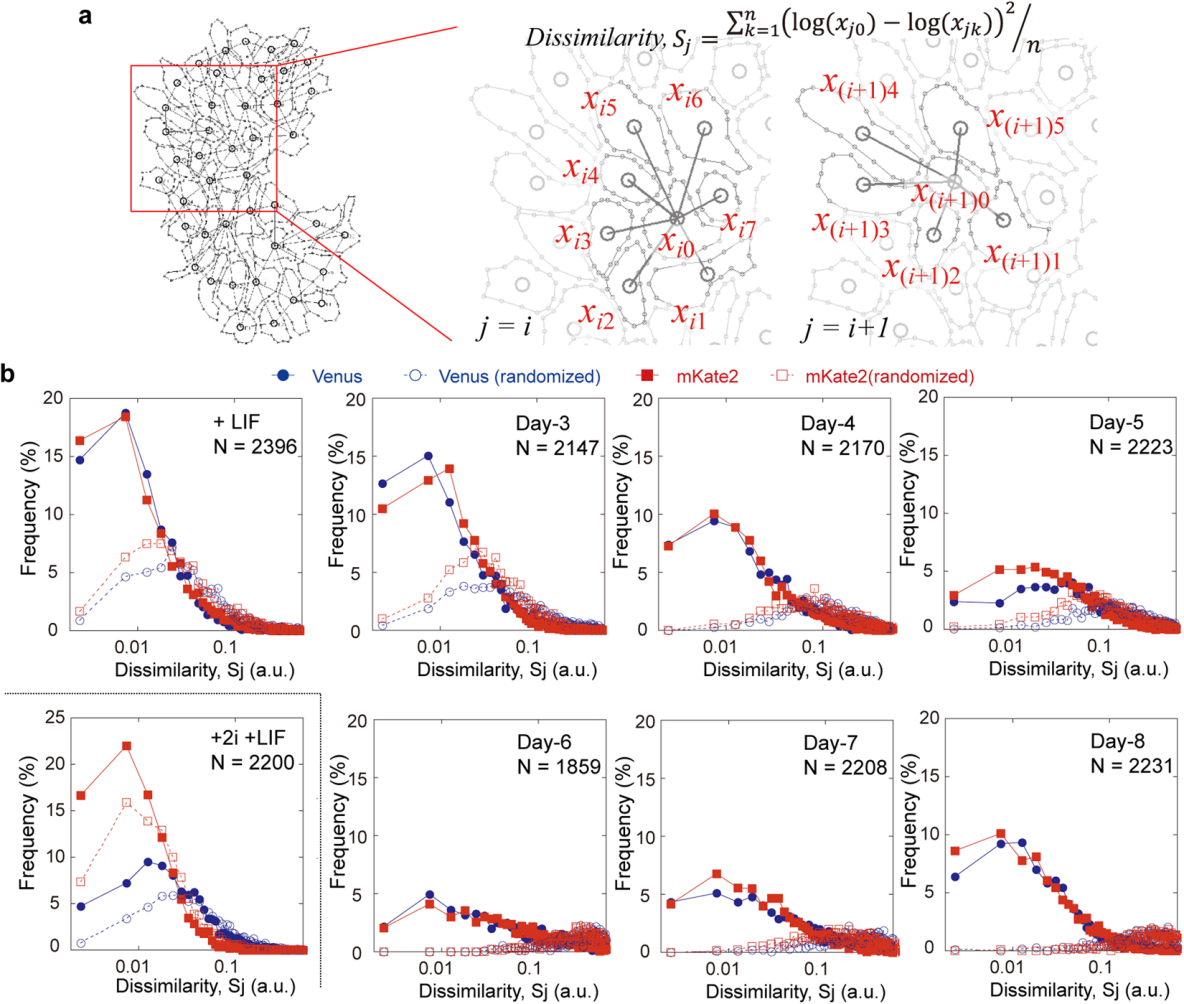
Measuring the gene expression variation through a dissimilarity metric. (**a**) Schematic explanation and equation of the ‘dissimilarity’ metric, defined as the summation of the mean square difference between a centre cell and all adjacent cells over the number of cells, when the centre cell had at least 4 adjacent cells. (**b**) Plots of the dissimilarity value in each culture condition. Solid symbols represent the experimental data of the Venus (blue circle) or mKate2 (red rectangle) fluorescence, while open symbols show the simulated data assuming that cell positions are randomized and that no cell-cell interactions occur.

Some cooperativity among the cells within each colony is necessary to generate the above bimodal response observed at the colony level (Fig. 2). In other words, to preserve the diversity at higher layers, some cooperativity is needed at the lower layer. Meanwhile, the cell-cell cooperativity should prevent the state transition driven by the intrinsic fluctuation. The balance of the cell-cell cooperativity and the intrinsic fluctuation would affect the variation in gene expressions. To evaluate the gene expression variation affected by the cooperativity, we defined a ‘dissimilarity’ metric according to the summation of the mean square differences between the logarithm of the fluorescence intensity of a given cell and that of all adjacent cells, divided by the number of the cells (Fig. 3a). Examples of this dissimilarity analysis are given in Fig. S8. Briefly, when within a single colony the fluorescence intensity of mKate2 was quantitatively more disparate among adjacent cells compared to the difference in the intensity of Venus reporter, higher dissimilarity values are obtained (Fig. S8, middle panels). In addition, a mixture of fluorescent cells and dark cells are represented by higher dissimilarity values (Fig. S8, right panels).

The dissimilarity values calculated for all groups of cells among all colonies increased for both Venus and mKate2 from Day-3 to Day-6, and then decreased (Fig. 3b, solid). To assess whether this behaviour is coincidental, experimental values were compared with artificial dissimilarity values calculated from the assumption that cells are randomly positioned (Fig. 3b, open). Compared to the randomized dissimilarities, the experimental values were significantly lower, with the exception of the +2i culture condition (i.e., in the presence of the MAPK/ERK and GSK3 inhibitors PD0325901 and CHIR99021, respectively), which is known to inhibit the differentiation signals^32^. The experimental dissimilarity exhibited an increase-decrease behaviour with increasing differentiating days (Fig. 3b, solid), while the randomized dissimilarity only followed a unidirectional increment over time (Fig. 3b, open). This quantitative analysis demonstrates that the fluorescence of Venus/mKate2 of a given cell was markedly similar to that of the surrounding cells, and the similarity temporally decreased at the intermediate stage of the differentiation process.

### Quantification of gene expression independence in a colony

**Figure 4:**
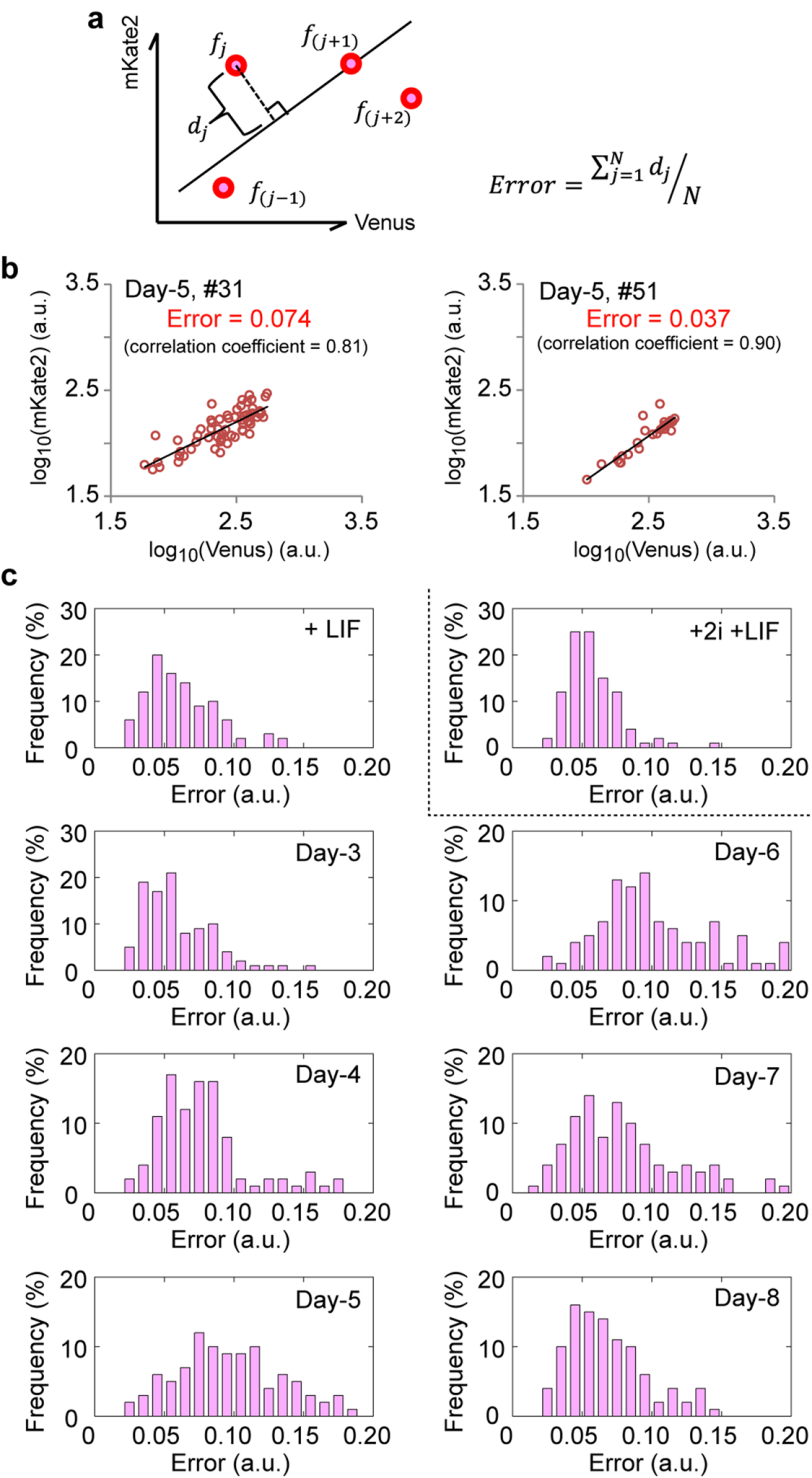
Measuring the degree of gene expression independence through an error metric. (**a**) Schematic explanation and equation of the ‘error’ metric. The ‘error’ metric is defined as the average of the distances *d*_*j*_ between each point and a fitting line defined by least-square method for a Venus and mKate2 correlation plot *f*(*j*). (**b**) Typical examples of the error analysis. Although the two colonies #31 (left) and #51 (right) at Day-5 exhibited similar mean intensities (150 a. u. for mKate2 and 285 a. u. for Venus in #31, and 123 a. u. for mKate2 and 334 a. u. for Venus in #51, respectively) and correlations, their error values were different. (**c**) Histograms of the error values in various culture conditions.

Nanog expression is usually well correlated with Oct4 expression^4,5^. The balance of the cell-cell cooperativity and the intrinsic fluctuation would also affect this correlation or independence in gene expressions. To evaluate the expression independence of each gene, we plotted Venus or mKate2 expression corresponding to Nanog or Oct4 expression level in each single cell of a given colony. Then, by fitting a line on the fluorescence values, we defined an ‘error’ metric as the average of the distances between plots and the line (Fig. 4a). Even when two colonies exhibited similar mean fluorescence intensities and a similar Venus/mKate2 correlation value, the evaluation of the error values of each colony revealed strong differences (Fig. 4b). When applied to all colonies, the error values increased from Day-4 to Day-6 and then decreased and the histograms of the error values showed a broad distribution at Day-5 and Day-6 (Fig. 4b), indicating that the fluorescence intensities of Venus and mKate2 had less correlation and greater independence.

### A toy model based on two fundamental roles hypothesized by the single-cell data

**Figure 5:**
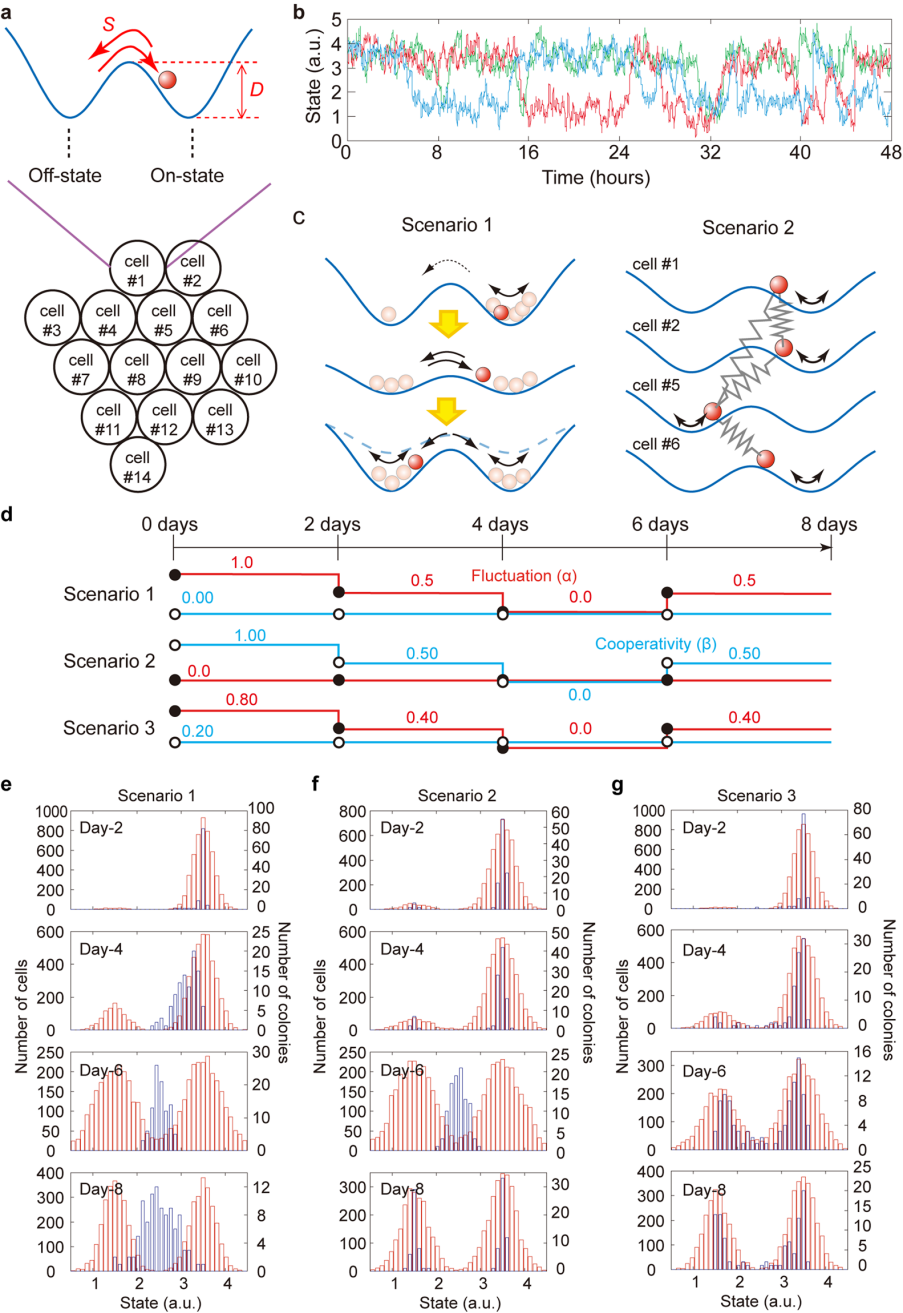
Toy model of the collective cell-state transition considering intrinsic fluctuation and cell-cell cooperativity. (**a**) Schematic of the model configuration. Any cell of the colony (hexagonal grid) can be described as a ball fluctuating on a potential landscape with two attractor states. (**b**) Example of three individual cells (coloured lines) switching randomly between the on-state and off-state. A Gaussian noise was applied to each cell behaviour to represent intrinsic fluctuation. (**c**) Schematic illustration of the cell-state transition driven by intrinsic fluctuation (left) or restrained by cell-cell cooperativity (right), here illustrated as springs connected to a ball, that are moving on a potential landscape. (**d**) Timing of parameter changes to perturb the fluctuation (red) and the cooperativity index (blue) for the three scenarios. (**e**–**g**) The behaviour of 100 colonies was simulated for each scenario and culture condition to monitor the dynamics of the cellular states of individual cells (red) and colonies (blue) in the absence of LIF.

The above results clearly showed that the variation and independence of the Venus/mKate2 expression, as measured by the dissimilarity and the error metrics, respectively, changed according to the same temporal pattern during the early differentiation stage. However, since we analysed only the observable expression patterns as a result of the cell-cell cooperativity and the intrinsic fluctuation, it is still unknown how these two factors each actually participate in the collective-state transition behind the scenes. To simply understand a basic mechanism of the observed behaviour by removing the unnecessary details, we constructed a numerical toy model to characterize the respective contribution of each property to the collective differentiation and the emergence of the bimodal distribution of the population observed at both the cell (around Day-6) and colony level (at Day-8) (Fig. 2c). One assumption based on previously published work was that a given cell can fluctuate between two states, ‘on’ and ‘off’ states^26–28^. This can be represented by a ball fluctuating back and forth between one valley (i.e., attractor state) and another (Fig. 5a, upper), a metaphor commonly used in physics^9,33^. In our model, each cell has a 50% probability of exhibiting one state or the other by Gaussian noise, described by the Langevin equation, independently of the other cells (Fig. 5b). The degree of intrinsic fluctuation was determined by the depth of the attractor (*D*) and the strength of the Gaussian noise (*S*) (Fig. 5a, upper), and the transition rate between two states is theoretically promotional to exp(−*D*/*S*). We here varied the degree of the intrinsic fluctuation by changing the attractor depth. The cells were set up on a hexagonal lattice in cellular automata (Fig. 5a, lower). Moreover, we assumed that a given cell can cooperate with contacting adjacent cells (for example, cell #5 communicates with cells #1, #2, #4, #6, #8, and #9 in Fig. 5a). The model allowed the cells to proliferate, and our simulation procedure took into account the passaging of culture cells (Fig. S9). It was also assumed that cell divisions randomly occurred every 11–13 hours, and that a given daughter cell was pushed forward in a random direction, when the daughter cell was surrounded by other cells. A total of 100 trials were performed for each culture condition (Supplementary Movies S1–S4).

We prepared three scenarios taking into account the intrinsic fluctuation and cell-cell cooperativity (Fig. 5c and d). In scenario 1, the cell-cell cooperativity is null while the degree of fluctuation reduces and then recovers, suggesting that the degree of the intrinsic fluctuation allows for the cell-state transition (Fig. 5c, left, and 5d, top). In scenario 2, the fluctuation is fixed to a constant value while cooperativity reduces and then recovers, which we illustrated as balls connected with springs whose stiffness was reduced and then recovered (Fig. 5c, right, and 5d, middle). Thus, the degree of cell-cell cooperativity alters the state transition of the neighbouring cells. In scenario 3, the cell-cell cooperativity is statically fixed to a given value while the fluctuation reduces and then recovers (Fig. 5d, bottom).

The first two scenarios were able to reproduce the appearance of the bimodal population at Day-6 at the single-cell level (Fig. 5e and f, red). However, at the colony level, the bimodal population appearing at Day-8 could only be seen in scenario 2 (Fig. 5f, blue), indicating that cell-cell cooperativity is necessary for colonial heterogeneity. In scenario 3, the population behaviour at the colony level followed the same dynamics as those observed at the single-cell level without any delay (Fig. 5g). When both fluctuation and cooperativity dynamically changed, although the experimental behaviour could be reproduced, the reproducibility depended on the degree of cooperativity but not on that of the fluctuation (Fig. S10). Thus, to reproduce the experimental results and observe the aforementioned delay between the single cells and the colonies at Day-6 and Day-8 (Fig. 2c), the dynamic change of the degree of cooperativity, rather than that of the fluctuation, is crucial. From the point-of-view of the potential landscape, the attractor depth for a cell to sense its environment changes in a deep-shallow-deep manner.

## Discussion

**Figure 6:**
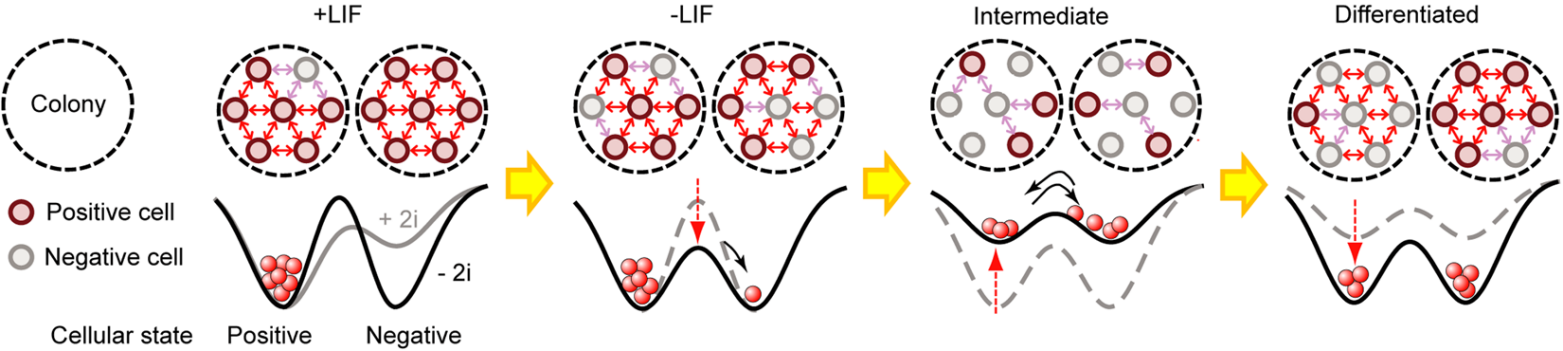
Illustration of the present working model based on the cooperativity of cells. Cells intrinsically fluctuate between the positive (red) and negative (gray) states. In the presence of LIF, cell-cell cooperativity restricts the fluctuations, and the energy barrier for the state transition becomes too high to be overcome; thus, mESCs stably remain in the positive state, i.e., pluripotent. In the absence of LIF signalling, the barrier is lowered, allowing some cells to stochastically transit to the other state. At the intermediate state, the attractor depth is decreased with the loss of cooperativity, and cells freely fluctuate between the two states. At the later stage, cells are attracted to either state by the reappearance of cooperativity. The attractor state, whose status is monitored by individual cells, dynamically changes in a deep-shallow-deep manner.

In light of our results, a working model is proposed as shown in Fig. 6. Our first assumption was that individual cells show inherent heterogeneous expression of their core transcription factors, which causes spontaneous intrinsic fluctuations from one state to another, the so-called ‘on’ and ‘off’ attractors previously reported in the literature^27,28^. Our second assumption was that this transition is restricted by some kind of cell-cell cooperativity, which is sensed by adjacent cells. We hypothesized that, in the presence of LIF, the cell-cell cooperativity restricts the intrinsic fluctuations so that all cells are in a similar pluripotent state (Fig. 6, +LIF). In other words, from a physical perspective, the energy barrier to be overcome to allow a cell-state transition is too high under this condition. In the absence of LIF signalling, the cell-cell cooperativity slowly decreases, allowing the cells to fluctuate stochastically and, rarely, transit to the other state (Fig. 6, −LIF). At the intermediate state, the progressive loss of cooperativity results in a shallower attractor depth so that the cells can more freely fluctuate between the two attractors (in our study, around Day-6; Fig. 6, Intermediate). At the later stage, the reappearance of cell-cell cooperativity prevents the cell-state transition. Based on the above working model, the cells are spontaneously and intrinsically fluctuating and the cell-cell cooperativity defines the attractor shape on the landscape that the individual cell rides on. Importantly, the depths of the attractor that finally a cell is sensing change in a deep-shallow-deep manner as a result of the change in cell-cell cooperativity (Fig. 6, Differentiated). This process is similar to a Brownian flashing ratchet mechanism^34^.

Historically, our understanding of stem cell biology has been enriched by the perspectives provided from various research areas. Fifty years ago, Till and colleagues took advantage of a mathematical model originally used to describe cosmic ray showers to demonstrate that some aspects of stem cell dynamics followed a stochastic process^35^. Likewise, to improve our understanding of stem cell biology, general mathematical models have been established based on the perspectives of ecological and evolutionary biology^36^ and systems biology^21^. We here propose that the collective cell-state transition follows the flashing ratchet-like behaviour. The flashing ratchet behaviour has been observed at various biological levels and scales such as molecular motors, mitochondrial protein import, cell migration, and cell shaping^37–41^. Even object recognition in brain could be explained by the similar model^42^. Ratchets can be seen as controllers that act on stochastic systems with the aim of inducing directed motion through the rectification of fluctuations^34^, and flashing ratchets drive the on-and-off switching of the periodic potential of particles^37,38^.

The state of the colony fluctuates in a similar fashion to that observed for individual cells, indicating that the heterogeneity is fractally structured over multilayers, which possibly maintains the diversity over multilayers. In other words, a homogenous group of self-decision-making cells will form a heterogeneous group through the action of fluctuation. In our opinion, a set of fluctuating individuals and their communication might be a fundamental element to generate the fractal structure in fluctuation, and the flashing ratchet behaviour consequently appears as an observable phenomenon. External factors biasing the bimodal pattern of distribution are also needed to generate the unidirectionality or asymmetry^37^. In the case of *in vitro* mESC differentiation, the addition of extracellular retinoic acids causes the cells to move away from their pluripotency state, while the addition of 2i/3i obliges the cells to remain in this state^32,43^.

Our toy model could demonstrate that the cell-cell cooperativity was particularly crucial to determine the cell fate at the colony level and could faithfully reproduce our experimental results. The simulation in which the parameters were constant during differentiation impressed the role of each parameter in our model (Figs S11 and S12). In the absence of the cooperativity, the depth of the attractor (parameter α) determining the degree of the intrinsic fluctuation was simply responsible for the rate of the state transition, and the population of the colony did not exhibit the bimodal distribution (Fig. S11). The strength of the spring (parameter β) determining the degree of the cell-cell cooperativity also altered the state transition rate, indicating that cells restricted the state transition of the neighbouring cells (Fig. S12). The bimodal distribution in colonies appeared as a result, and the degree of the cooperativity needed to be sufficiently small for the bimodal distribution to disappear (Fig. S12, blue). Furthermore, even with numerous tested combinations of the two parameters, the experimental behaviour, the disappearance and appearance of bimodal distribution in colonies, could not be reproduced as long as the parameters were statically fixed (Fig. S13). Therefore, the dynamic change of the parameters during the differentiation was fundamental to reproduce the collective behaviour we observed here.

The collective behaviour of mESCs implies that the cells can sense the status of closely located cells and react accordingly. The molecular mechanism under the collective behaviours of mESCs has been well investigated as mentioned in the introduction. Also, another possible mechanism that may contribute to cell-cell cooperativity is the role played by mechanical stress, such as cell ‘stiffness’ or cell-cell adhesion. For example, the cell-cell cooperativity mediated by E-cadherin might contribute to produce the asymmetry of the bimodal distribution, because the E-cadherin antibody addition into the medium maintains the pluripotency state without colonizing^44^. It was also reported that the actin cytoskeleton is correlated to Nanog expression in mESCs through the PI3K/AKT signalling cascade^45^, and that the softness of cell-cell adhesions promoted Nanog and Oct4 expression by inhibiting actin polymerisation^46^. The very intricate relations between paracrine signalling, cell-cell adhesion and actin polymerisation seem to define a complex regulatory network that guides the activity of core transcription factors during the differentiation process of stem cells.

Although we did not quantify the properties of cell adhesion in this study, we observed that in the presence of 2i the adhesion of cells was hindered, and cells could not settle properly (which is why we used Matrigel for this particular condition, see Methods). Based on this observation, we speculate that the coated surface and cell adhesion may have affected the collective behaviour of cells. In fact, the addition of 2i in the medium decreased the variation index, as indicated by the small difference in the dissimilarity index between the artificial (i.e., randomized) and experimental values (Fig. 3b, +2i +LIF) by comparison to the other conditions. However, the Nanog-Oct4 correlation was the highest among the examined conditions (Fig. 4b, +2i +LIF). One possibility is that the 2i restricted the intrinsic fluctuation in mESCs to stabilize the pluripotent state without interacting with surrounding cells. This hypothesis is supported by a previous literature reporting that the addition of 2i inhibited the fluctuation of the morphology of mES colony between the round to the flatten form in the presence of LIF^47^. Another possibility is that the surface coating could regulate Oct4 expression as suggested in the literatures^46,48^. Both factors likely contribute to the observed behaviour. In the future, we aim to unveil the extent and molecular basis of this intricate network and its role in defining cell-cell cooperativity and cell-state transitions.

More generally, collective behaviours have a long history in various research fields, including physics, social sciences, and biological sciences^49–53^. Bird flocking, fish schooling, and insect swarming are well-known examples of collective behaviours of organisms, whose mechanisms such as density-dependent thresholds have been elucidated based on physical and mathematical models^51^. This study found that mESCs cooperate with each other, and such cooperation was crucial to drive the emergence of a bimodal distribution at both the single-cell level and colony level by regulating the intrinsic fluctuations. This specific mechanism could be explained by a simple toy model based on the Langevin equation (Fig. 5), which has been widely used to describe the motion of Brownian particles moving in a flashing periodical potential^37–41^. Interestingly, this equation is composed of the terms of fluctuation and communication (see Methods), and has been widely used to describe collective behaviours in flocking birds^54^ and human society^55^. In cases for which an individual can choose between two alternative responses, it was shown that at the group level, individuals behave using collective decisions as the result of communication. Other collective behaviours of living cells including the cell migration observed during morphogenesis, tumour invasion, wound healing and so on, have been well studied, and these collective actions of individual cells are thought to be guided by density-dependent signals, complex chemical feedback, mechanical cues, and leading cells^56^. In this regard, it is worth mentioning that by contrast to other collective behaviour, mESCs do not follow leading cells or oscillators. No circadian clock is present, even in the pluripotent state^57^. By contrast, our study found that mESCs can different in a collective manner. The collective behaviours in mESCs we observed here might be an inherent and common denominator of the decision-making beyond consideration of scale and species in biology.

It was thought that continuous time-lapse imaging was not a suitable choice for our study. In sequential image acquisition, a time resolution of about 15 min was required to monitor cell division. In a preliminary experiment using 15 min time-lapse imaging of mESCs, we found that the repeated laser illumination strongly compromised the survival of the cells after 48 h^58^. Moreover, during our snapshot experiments (Figs 1 and S1), we found that the cells were more sensitive to laser illumination at a certain stage of differentiation, especially during the intermediate stage, from Day-4 to Day-7. Another option could have been to avoid the use of the laser and fluorescence, and rely instead on luciferase reporters^59^. This system, developed recently, could be an interesting tool, but was not applicable to our research because the concentration of the exogenous substrates, i.e., coelenterazine, and their distribution within cells are uncontrollable, which would reduce the ability for quantitative analyses of the fluorescence intensities.

In conclusion, the present study unveiled the process of fractal heterogeneity (i.e., at the cell and colony levels) during the collective differentiation of mESCs. Although it remains unclear whether such heterogeneity in mESCs is a driving force for cell fate commitment^60^, at this point we can conclude that the spontaneous fluctuation of protein expression is an intrinsic and necessary feature to guide the state transition in a cell, and that cell-cell cooperativity exerted at the group level, plays a role in restricting this fluctuation. By combining these two factors, flashing-ratchet behaviour could be defined to establish a general analogical mechanism driving the collective decision-making during cell differentiation. The flashing ratchet model may provide a better understanding of the collective dynamic processes in stem cells and other oscillating cell populations.

## Methods

### Establishment of stable cell lines

Mouse fluorescent reporters were constructed using a pRedZeo-lenti pluripotency reporter system (System Bioscience, USA, SR10044PA-1 and SR SR10045-PA-1). A Nanog-Venus reporter was constructed by adding BamHI/SalI sites to the cDNA of a Venus fragment by PCR amplification, and the fragment was replaced by the gene encoding red fluorescent protein (RFP) of a SR10044-PA-1 plasmid. To construct an Oct4-mKate2 reporter, the cDNAs of an Oct4 reporter containing Oct4-RFP (SR10045-PA-1) and mKate2 fragment were amplified by PCR containing ClaI/BamHI sites and BamHI/Sal I sites, respectively. The constructs were then transformed into *Escherichia coli* DH5α. Plasmid purification was performed using HiPure plasmid Filter Midiprep Kit according to the manufacturer’s protocol (Invitrogen, USA, K210015).

The plasmids mentioned above were transfected into 293T cells with FuGENE HD (Promega, USA, E231A) to produce lentivirus. Mouse embryonic stem cells (mESCs; Riken Cell Bank, JP, E14Tg2a cell line)^61^ were transfected with the lentivirus encoding a single reporter. Reporter-positive mESCs were isolated using fluorescence-activated cell sorting (FACS; BD Biosciences, USA, BD FACS Aria III™). We repeated these steps to obtain two reporter-positive mESC lines.

### Experimental design

The experimental plan is described in Fig. S1. The aim of our experiment was to follow the protein expression levels on a daily basis at both the single-cell and colony levels. Preliminary experiments showed that time-lapse imaging was not suitable for reasons we describe in the Discussion. Thus, we opted for dividing an initial culture into two sequential sets: one harvested at Day-3, Day-5, and Day-7 of differentiation, and the other harvested on Day-4, Day-6, and Day-8 of differentiation. The two-day intervals were required to grow and passage the cells. To establish the initial culture, 1 × 10^5^ cells were seeded on 10-cm plastic dishes (BD Biosciences, 353003) coated with EmbryoMax® 0.1% gelatin (Merck Millipore, Germany, ES-006-B). At each passage, the cells were divided into two plates, one for microscopic observation and the other for FACS analyses and new passages (Fig. S1). For microscopic observation, 1 × 10^5^ cells were seeded on 35-mm glass bottom dishes (Matsunami-Glass, Japan, D11130H) coated with 0.1% gelatin solution. It is worth mentioning that the cells could not be fixed properly in the +2i +LIF culture condition, and so we used 1:40 diluted BD Matrigel™ (BD-Biosciences, 354277) instead, which has a potential impact on the result as described in the Discussion. We observed no significant variations in terms of cell shape, colony size, or gene expression levels between the dishes used for fluorescence imaging or FACS analyses (Figs 1, S2 and S3).

### Reagents for cell cultures

Established cell lines were cultured and maintained in Dulbecco’s modified Eagle medium (DMEM; Sigma–Aldrich, USA, D6046) containing 10% foetal bovine serum (FBS; Gibco, USA, 16141–075), 1% penicillin-streptomycin (Sigma–Aldrich, P4333), 1% GlutaMAX-1 (Gibco, 35050–001), 1% non-essential amino acids (Gibco, 11140–050), 1% nucleosides (Millipore, USA, ES-008-D), 1% sodium pyruvate (Sigma–Aldrich, S8636), 0.1% 2-mercaptoethanol (Sigma–Aldrich), and 0.1% leukaemia inhibitory factor (LIF; Nacalai, Japan, NU0013–1) on 0.1% gelatin-coated 10-cm dishes (BD Biosciences, 353003) without feeder layers. In the +2i +LIF culture condition, the cells were cultured using DMEM with 10% knock out serum (KSR; Gibco, 10820–028) instead of 10% FBS, and supplemented with the inhibitors to MAPK and GSK3 using PD0325901 (Stemgent, USA, Stemolecule™ 04–0006) and CHIR 99021 (Stemgent, Stemolecule™ 04–0004) at 1 µM and 3 µM, respectively. Cells were passaged every 2 days.

### FACS analysis of single cells

The attached mESCs were harvested by trypsinization, and filtered according to the manufacturer’s protocol for FACS, and suspended in DMEM with the supplements described above. The cells were then analysed using BD FACS Aria III with BD FACS Diva software (BD-Biosciences, BD FACS Aria III™). Gates of the size, volume, and background signals of cells were set using non-transfected ESCs to ensure meaningful measurements. Venus, reporting Nanog expression, was excited by a blue (488 nm) laser, and filtered with a 515–545 nm bandpass filter, and mKate2 reporting Oct4 expression was excited by yellow-green (561 nm) laser, and filtered with a 600–620 nm bandpass filter. In the case of FACS with Nanog immunostaining using Alexa Fluor 647, excitation was used a red (633 nm) laser, and emission was filtered with a 650–670 bandpass filter.

### Microscope observation of colonies

Prior to observation, the cells were incubated with 500 nM DRAQ5™-supplemented medium (BioStatus, UK, DR50050) at 37 °C for 15 min for nuclear staining. Then, the medium was changed to phenol red-free DMEM (Gibco, 31053–028) with supplements. Fluorescence imaging was carried out with an inverted confocal microscope (FV1000, Olympus, Japan) combined with a stage-top incubator to control the temperature at 37 °C (INUC-KRi, Tokai-Hit, Japan). CO^2^ was loaded at 5% in the incubator (GM-2000, Tokai-Hit, Japan). Venus, reporting Nanog expression, was excited by a blue (473 nm) laser, while mKate2, reporting Oct4 expression and DRAQ5 were simultaneously excited by a green (559 nm) laser. Fluorescent signals were selected by a 405/473/559/635 multi-edge dichroic mirror, and divided into two pathways by a 560 single-edge dichroic mirror. Wavelengths below 560 nm were filtered with a band-pass filter from 490‒540 nm, and wavelengths above 560 nm were filtered by a 640 single-edge dichroic mirror, and separated with 575‒620-nm and 655‒750-nm band-pass filters. A 60× objective lens (NA 1.40, oil, PLAPON 60XOSC2, Olympus, Japan) and an observation area of 211.76 × 211.76 µm^2^ (1024 × 1024 pixels) were used. The high NA objective lens was needed to clearly identify the cell periphery. The pixel dwell time was 2.07 µs. One hundred ESC colonies were analysed for each time point and/or culture condition. In some cases, we observed some migrating cells departing from the colony (Fig. S14). These cells were not taken into account for quantification of the dissimilarity index and error analysis.

### Image analysis

We initially planned to perform the data analysis of fluorescence microscopy images by automatic selection of the cells according to their nuclear shape (stained with DRAQ5), using our home-made software. However, the nuclei of undifferentiated ESCs showed more complicated shapes and were larger than those of differentiated cells, which made the automatic identification of single cells difficult, if not impossible. Therefore, to ensure that no artificial variation corrupted our calculation of fluorescence intensities, we manually delimited each individual cell with a polygon, based on the information provided by the DRAQ5-stained nuclei and the transmission light microscope image (Fig. 2a, lower panels). Cell-cell contacts were also identified by eye to connect adjacent cells. The fluorescent intensity I of each cell was defined as the average value of the pixel intensities contained in the polygon (i.e., surface equivalent) after background subtraction (Fig. 2b). The mean fluorescent intensity in a colony (Figs 2d and S6) was calculated as the summation of the value of each cell divided by the total number of cells.

### Mathematical toy model simulation

Assuming that a given cell could fluctuate on a potential landscape with two attractors, a positive state (*x*_*P*_, 0) and a negative state (*x*_*N*_, 0), the potential shape of the landscape was represented with two quadratic functions with vertices (*x*_*N*_, 0) and (*x*_*P*_, 0), and coefficients *α*:

1 $$U(x,t)=\{\begin{array}{cc}a\cdot {(x-{x}_{P})}^{2} & x\ge \frac{{x}_{P}+{x}_{N}}{2}\\ a\cdot {(x-{x}_{N})}^{2} & x < \frac{{x}_{P}+{x}_{N}}{2}\end{array}\},$$

where *a* represents the depth of the attractors. We also assumed that a given cell located at position (*i*, *j*), *S*_(*i*,*j*)_, cooperates with the adjacent cells (i.e., in direct contact) at position (*p, q*), *S*_(*p*,*q*)_. We made this assumption so that the cooperativity could be modelled as a spring connecting cells to their potential landscape (Fig. 5c), *F*_(*i*,*j*)_, as defined by:

2 $${F}_{(i,j)}=k\cdot \sum ({S}_{(p,q)}-{S}_{(i,j)}),$$

where *k* represents the stiffness of the spring.

The fluctuation dynamics of the cellular state located at position (*i*, *j*) was described by the Langevin equation as:

3 $$\frac{d{S}_{(i,j)}}{dt}=-\,\frac{\partial U({S}_{(i,j)},t)}{\partial {S}_{(i,j)}}+{F}_{(i,j)}+\xi (0,\sigma ),$$

where *ξ*(*u*, *v*) is the Gaussian noise, *u* is the mean value, and *v* is the standard deviation. The parameters *a* and *k* were changeable as follows:

4 $$\begin{array}{c}a={a}_{0}+{a}_{1}\cdot \alpha \\ k={k}_{0}\cdot \beta ,\end{array}\,$$

where the variables α and β depend on the specific scenarios considered.

The simulation’s computations were performed with a home-made program. The calculation of the simulations was implemented using a home-made software program constructed using Visual Studio (Visual Studio 2010 Professional academic edition, Microsoft, USA) and OpenCV library (OpenCV2.2, BSD license). The Euler-Maruyama method^62^ was applied to approximate the numerical solutions of the stochastic differential equations. The Gaussian noise was determined by the Box-Muller’s method^63^. The sampling frequency of the calculation was 60 points per minute. We initially set four cells on the lattice in the hexagonal cellular automata. Cells randomly divided for 11–13 hours, and the cell number increased to about 30 cells after 172,800 points, which corresponds to 2 days of virtual culture. Virtual passages were performed every 172,800 points (2 days). At each cell passage, the arrangement of cells was shuffled, and a set of four new initial cells was randomly established. The constants were set as *x*_*N*_ = 1.5, *x*_*P*_ = 3.5, *a*_0_ = 0.03, *a*_1_ = 0.10, and *k*_0_ = 0.20. The initial value of *S*_(*i*,*j*)_ was set to 3.5. To determine the initial cells that constitute the lattice at each passage, we simulated 345,600 points corresponding to 46 hours with the parameters *a* = 1.0 and *k* = 0.20. The parameter settings for α and β are described in Figs 5d and S10–S13.

## Electronic supplementary material

Movie S1

Movie S2

Movie S3

Movie S4

Supplementary figures S1-S14

Appendix data table S1
