# Supplementary material for: Single cell analysis reveals a biophysical aspect of collective cell-state transition in embryonic stem cell differentiation
Source: Sci Rep. 2018 Aug 10;8:11965. doi: 10.1038/s41598-018-30461-2 (PMC6086879; doi:10.1038/s41598-018-30461-2)
Supplement: Supplementary file 5 — Supplementary figures S1-S14 [file 41598_2018_30461_MOESM5_ESM.docx]

**Supplementary Information**

**Single cell analysis reveals a biophysical aspect of**

**collective cell-state transition in embryonic stem cell differentiation.**

Kazuko Okamoto^1^, Arno Germond^1^, Hideaki Fujita^2^,

Chikara Furusawa^1,3^, Yasushi Okada^1,3,4^, Tomonobu M. Watanabe^1*^

^1^RIKEN Center for Biosystems Dynamics Research (BDR), 2-6 Furuedai, Suita, Osaka 565-0874, Japan

^2^WPI, Immunology Frontier Research Center, Osaka University, 3-1 Yamadaoka, Suita, Osaka 565-0871, Japan

^3^School of Science, the University of Tokyo, 7-3-1 Hongo, Bunkyo-ku, Tokyo 113-0033, Japan

^4^Graduate School of Frontier Bioscience, Osaka University, 1-3 Yamadaoka, Suita, Osaka 565-0871, Japan

This file includes

Supplementary Figures S1–S14.

**Supplemental Figures.**


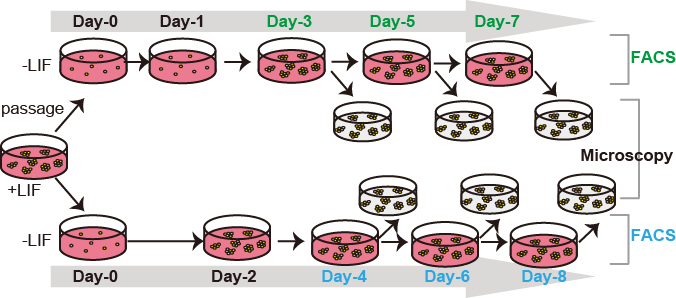


**Supplementary Figure S1. Procedure of the experiment.**

Cells were cultured in the presence of LIF (+LIF) to establish the initial culture. Then, cells were cultured in the absence of LIF (-LIF) to monitor the early stage of the differentiation process. At each time point, cells were either used for microscopic observation or to perform FACS analyses and passaging.


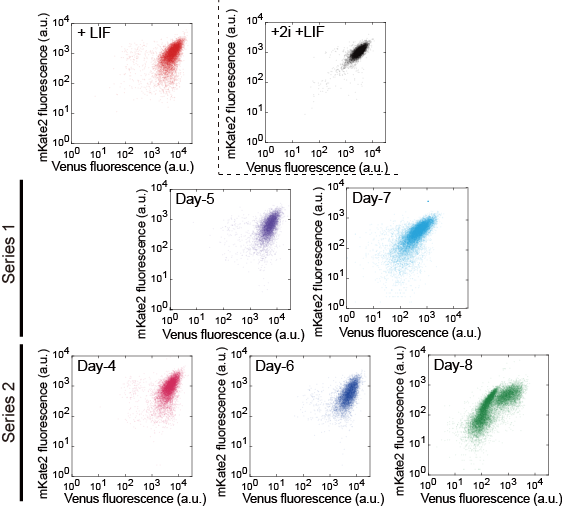


**Supplementary Figure S2. Characterization of mESC differentiation by flow cytometry.**

Correlation plots of Venus (reporting Nanog) and mKate2 (reporting Oct4) fluorescence measured in single cells in the presence of LIF signalling with (+2i +LIF) or without 2i inhibitors (+LIF) and from 4 to 8 days after removing LIF (Day-4 to Day-8). Vertical and horizontal axes represent the average fluorescent intensities of the mKate2 and Venus fluorescence reporters, respectively.


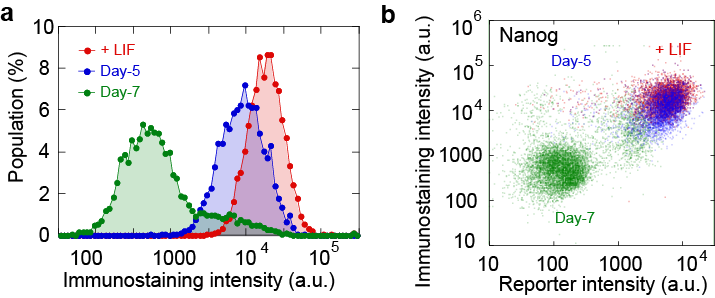


**Supplementary Figure S3. Comparison of fluorescent protein reporter system and immunostaining by flow cytometry.**

(**a**) Histogram of fluorescent intensity in immunostaining of Nanog analysed with a flow cytometer in the presence of LIF (+LIF, *red*) and at Day-5 (*blue*) and Day-7 (*red*) in the absence of LIF. (**b**) Correlation of fluorescent intensities of immunostaining and fluorescent protein reporter (Venus) of Nanog analysed with a flow cytometer in the presence of LIF (+LIF, *red*) and at Day-5 (*blue*) and Day-7 (*red*) in the absence of LIF. For the immunostaining, the cells were fixed with 4% PFA, permeabilised with 0.1% Triton-X, and then labelled with primary antibody to Nanog (abcam, UK, ab80892) and Alexa Fluor 647 conjugated 2nd antibodies (CST, Japan, #5264).

**
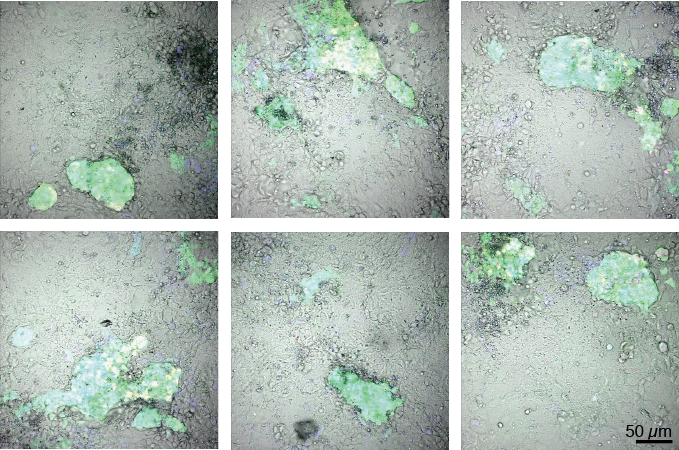
**

**Supplementary Figure S4. Observation of mESCs at Day-14 in the absence of LIF without passaging.**

The superimposed images of confocal fluorescence and transmission image of the mESCs at 14 days after removing LIF (Day-14) without passaging the cells. Over the long-term culture, the mESCs formed compact colonies that increased in size and eventually merged together. The colonies were composed of cells that exhibited high green fluorescence reporting Nanog expression.


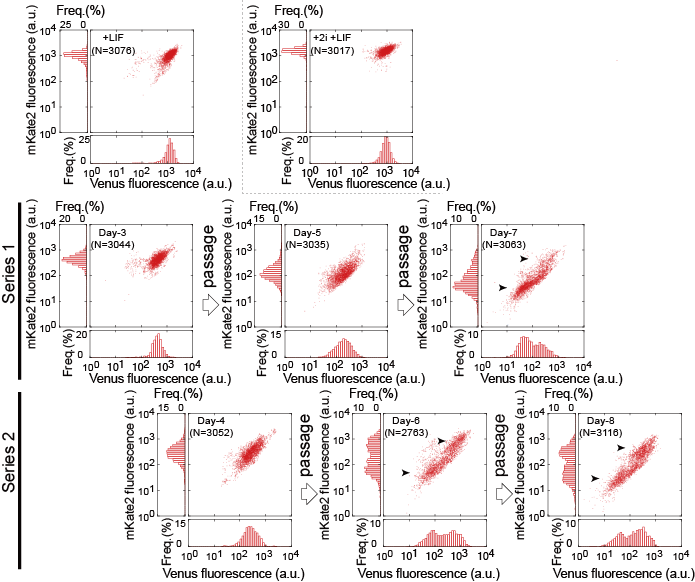


**Supplementary Figure S5. Single-cell analysis of the fluorescent intensities of Venus and mKate2.**

Correlation plots of Venus (reporting Nanog) and mKate2 (reporting Oct4) fluorescence measured in single cells in the presence of LIF with or without +2i inhibitors and in the absence of LIF from Day-3 to Day-8. Vertical and horizontal axes represent the average fluorescent intensities of the mKate2 and Venus fluorescence reporters in a cell, respectively. Left and bottom panels show the corresponding histogram distribution. Arrowheads indicate the presence of bimodal populations.


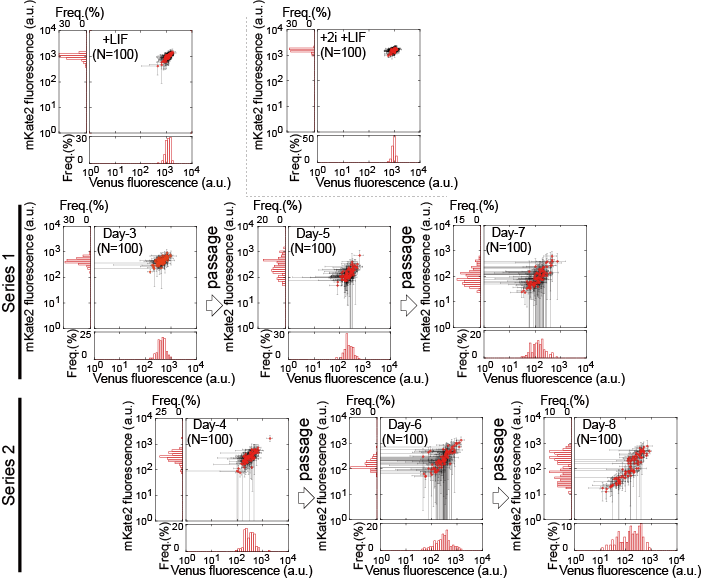


**Supplementary Figure S6. Single-colony analysis of the fluorescent intensities of Venus and mKate2.**

Correlation plots of the mean intensity of Venus (reporting Nanog) and mKate2 (reporting Oct4) fluorescence measured in single colonies in the presence of LIF with or without +2i inhibitors and in the absence of LIF from Day-3 to Day-8. Each point represents the average fluorescent intensities of all cells within a colony. Black lines indicate standard deviations in a colony. For each plot, the left and bottom panels represent histograms of the mKate2 and Venus fluorescence reporters, respectively.


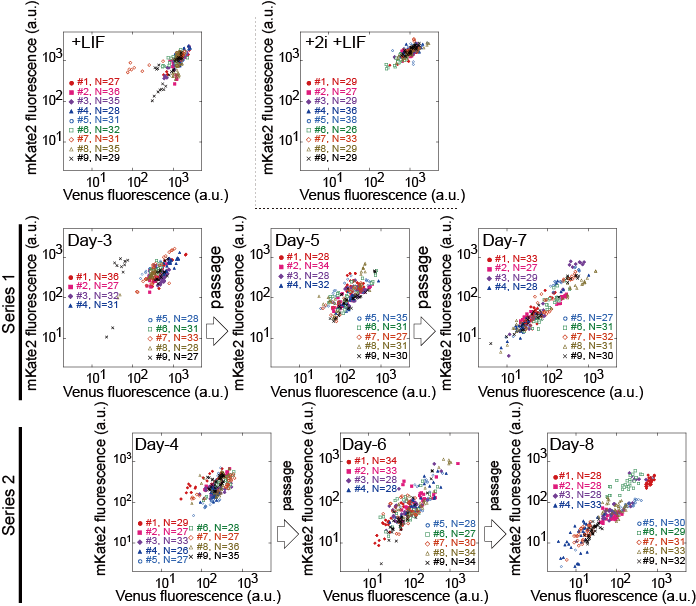


**Supplementary Figure S7. Multiscale heterogeneity observed in the Venus-mKate2 correlation.**

Correlation plots of Venus (reporting Nanog) and mKate2 (reporting Oct4) fluorescence measured for each cell within each colony in the presence of LIF with or without +2i inhibitors and in the absence of LIF from Day-3 to Day-8. Each colony is represented by a different symbol, and cells composing a colony are indicated in specific colours.

**
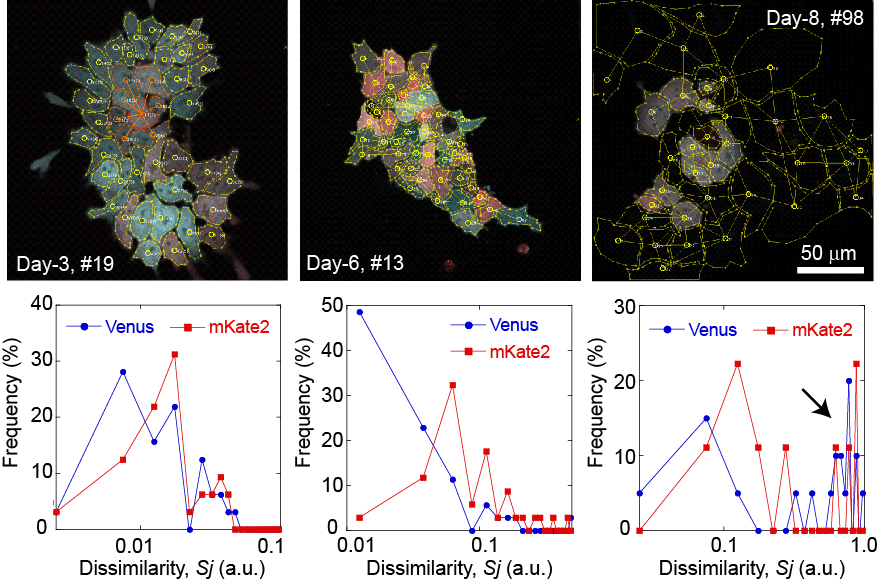
**

**Supplementary Figure S8. Representative examples of the dissimilarity analysis on three colonies.**

Images of fluorescence microscopy (upper) and the corresponding quantitative analyses of the dissimilarity index are shown for each reporter: Venus (reporting Nanog) in blue, and mKate2 (reporting Oct4) in red. The left picture is the colony presented in Fig. 2ab, and the corresponding analysis showed that Venus and mKate2 exhibit somewhat similar patterns of dissimilarity. The middle picture (Day-6, #13) and its analysis show a case in which Oct4 (red) expression is highly heterogeneous, as indicated by the increase in the dissimilarity frequency. Right panels (Day-8, #98) show a case in which almost all of the cells were differentiated, as indicated by the lack of reporter fluorescence. This resulted in strong dissimilarity values (an arrow) because of the large difference between the cells with no fluorescence and those with high reporter expressions.


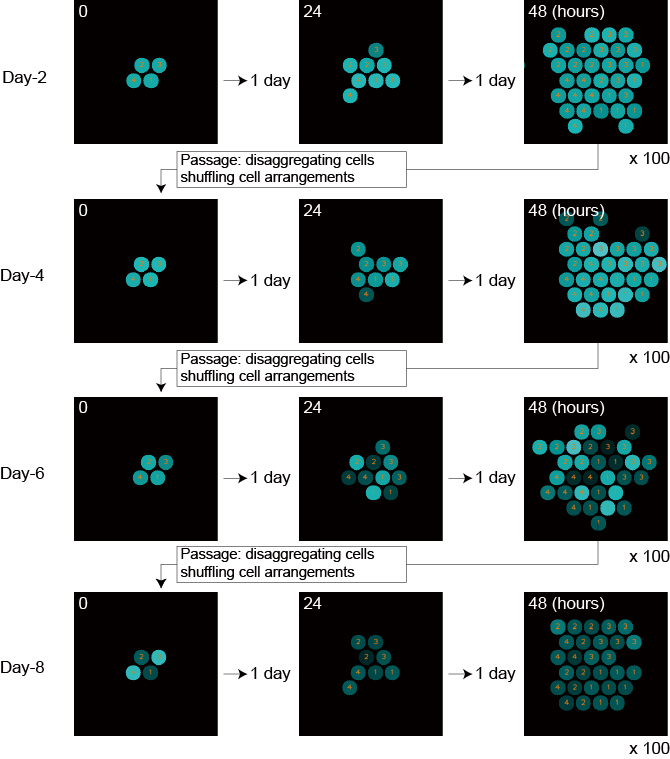


**Supplementary Figure S9. Schematic representation of the simulation procedure.**

At the initial time point, four cells were set on the lattice in hexagon cellular automata (left panels). The cells were allowed to randomly divide after 6–8 hours of growth. In one passage, 172,800 time points, corresponding to 2 days, were calculated. At each passage, the arrangement of cells was shuffled, and four new initial cells were used to initiate a new cycle. To simulate 100 colonies, a total of 100 cycles were performed.

**
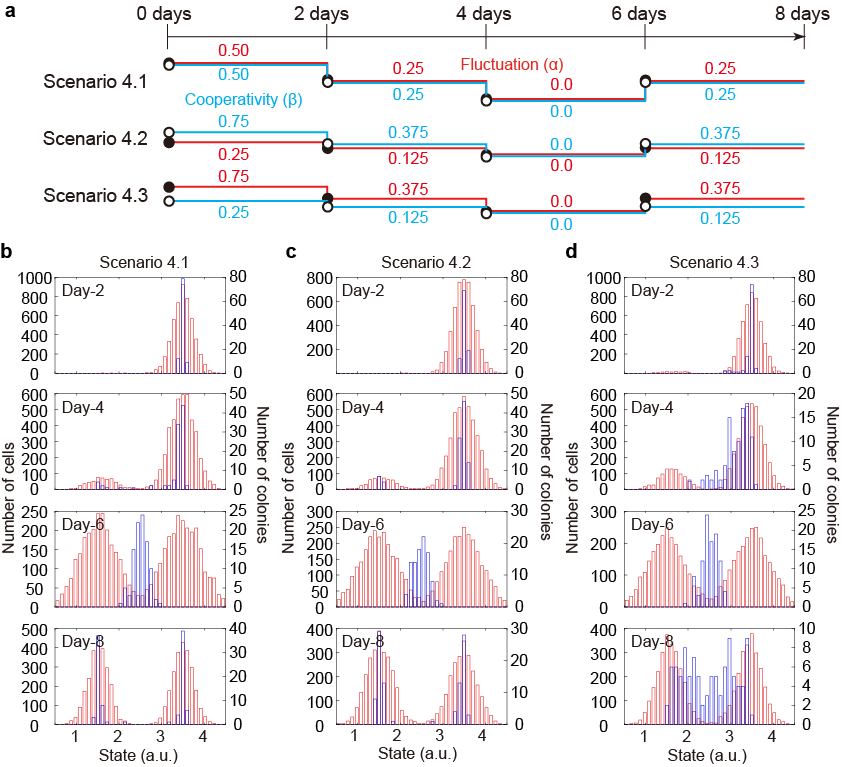
**

**Supplementary figure S10. Additional scenario in our toy model of the collective cell-state transition considering intrinsic fluctuation and cell-cell cooperativity.**

**(a)** Timing of parameter changes to perturb the fluctuation (red) and the cooperativity index (blue) for the three scenarios (4.1-4.3). **(b-d)** The behaviour of 100 colonies was simulated for each scenario and culture condition to monitor the dynamics of the cellular states of individual cells (red) and colonies (blue) in the absence of LIF.


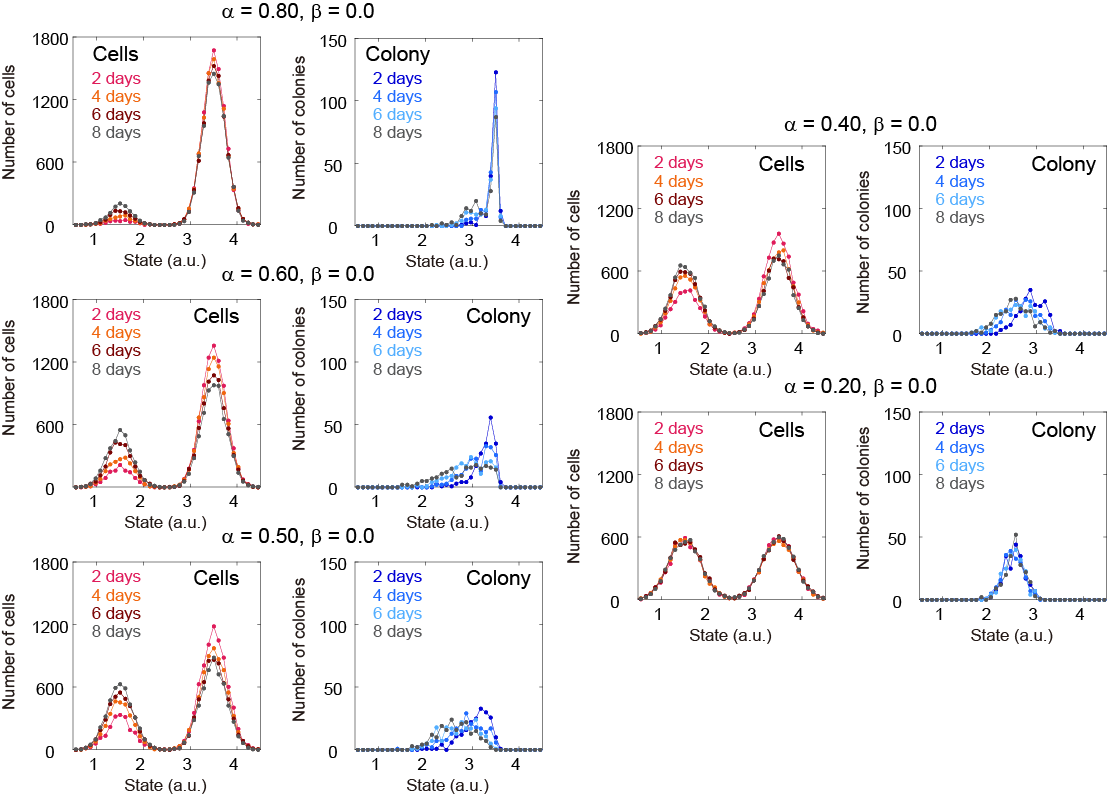


**Supplementary figure S11. Simulation result of our toy model when the parameter for the attractor depth (α) is statically fixed.**

The behaviour of 200 colonies was simulated to monitor the dynamics of the cellular states of individual cells (red) and colonies (blue) at the various value of the parameter α as the parameter for the spring strength (β) was constantly zero.


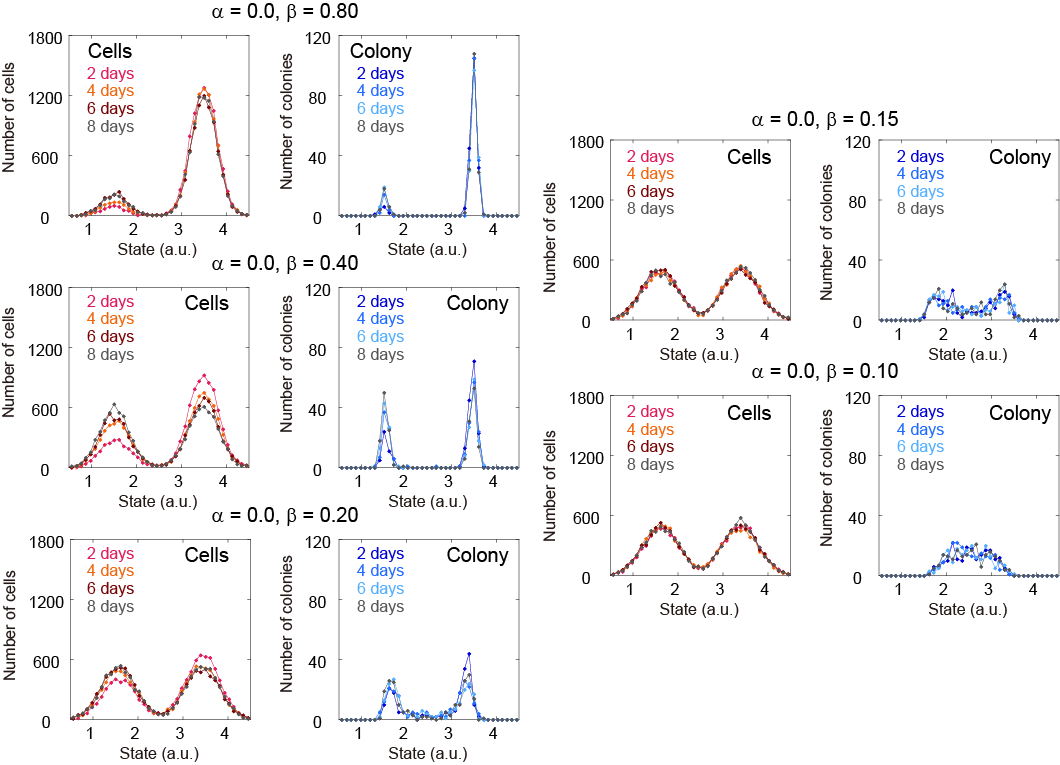


**Supplementary figure S12. Simulation result of our toy model when the parameter for the spring strength (β) is statically fixed.**

The behaviour of 200 colonies was simulated to monitor the dynamics of the cellular states of individual cells (red) and colonies (blue) at the various value of the parameter β as the parameter for the attractor depth (α) was constantly zero.


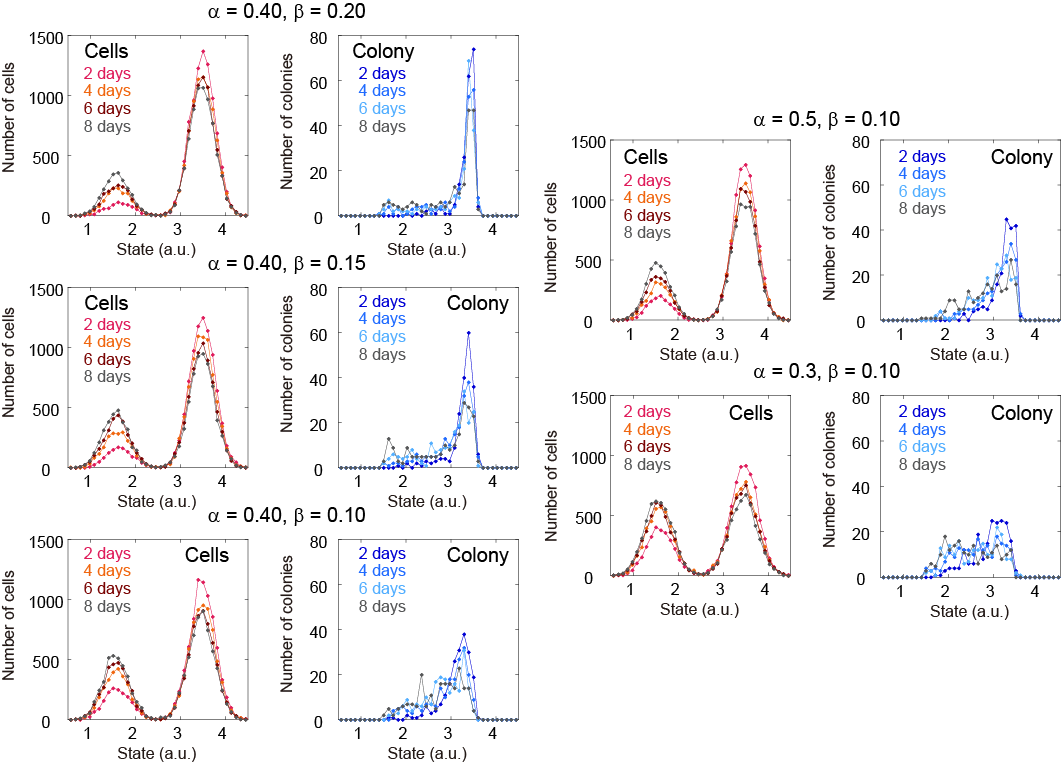


**Supplementary figure S13. Simulation result of our toy model when the parameters for the attractor depth (α) and the spring strength (β) are statically fixed.**

The behaviour of 200 colonies was simulated to monitor the dynamics of the cellular states of individual cells (red) and colonies (blue) at the various values of the parameter α and β.


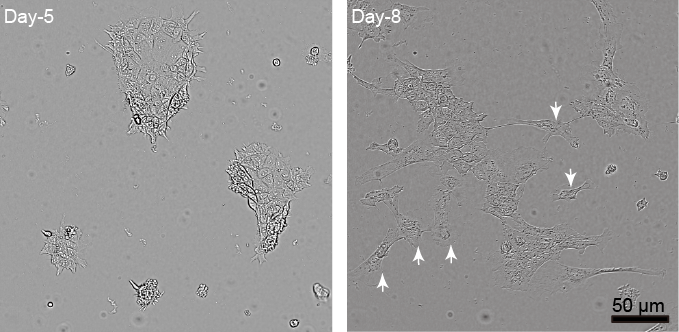


**Supplementary Figure S14. Cell behaviours in the differentiated state of the established mESC line.**

Low-magnification microscope images of colonies at Day-5 and Day-8 in the absence of LIF. In the left picture, the shape of the colony was well maintained. In the right picture, after Day-8, the shape of the colony was no longer circular, which we explain by the appearance of differentiated cells that spread and migrated out from the colonies as indicated by white arrows. We did not take into account these isolated cells for the analyses.
